# Toward Rational Design of Novel Anti-Cancer Drugs Based on Targeting, Solubility, and Bioavailability Exemplified by 1,3,4-Thiadiazole Derivatives Synthesized Under Solvent-Free Conditions

**DOI:** 10.3390/molecules24132371

**Published:** 2019-06-27

**Authors:** Huda R.M. Rashdan, Mohammad M. Farag, Marwa S. El-Gendey, Marwa M. Mounier

**Affiliations:** 1 Chemistry of Natural and Microbial Products Department, Pharmaceutical and Drug Industries Research Division, National Research Centre, Dokki, Cairo 12622, Egypt; 2 Glass Research Department, National Research Centre, 33 El-Behooth Str., Dokki, Cairo 12622, Egypt; 3 Chemistry Department, Faculty of Science “Girls”, Al-Azhar University, Cairo 11754, Egypt; 4 Chemistry Department, Faculty of College, Turabah, Taif University, Taif 21974, Saudi Arabia; 5 Pharmacognosy Department, Pharmaceutical and Drug Industries Research Division, National Research Centre, Dokki, Cairo 12622, Egypt

**Keywords:** green chemistry, 1,3,4-thiadiazoles, 1,2,3-triazoles, anticancer activity, microspheres, drug delivery

## Abstract

The 1,3,4-thiadiazole derivatives (**9a**–**i**) were synthesized under solvent free conditions and their chemical composition was confirmed using different spectral tools (IR, Mass, and NMR spectrometry). All the synthesized compounds were screened for their anti-cancer potentiality over human breast carcinoma (MCF-7) and human lung carcinoma (A-549). Most of the tested compounds showed remarkable anti-breast cancer activity. However, compound **4** showed the most anti-lung cancer activity. Then, compounds with cytotoxic activity ≥ 80% over breast and lung cells were subjected to investigate their specificity on human normal skin cell line (BJ-1). Compounds **9b** and **9g** were chosen owing to their high breast anti-cancer efficacy and their safety, in order to study the possible anti-cancer mode of action. Otherwise, drug delivery provides a means to overcome the low solubility, un-targeted release, and limited bioavailability of the prepared 1,3,4-thiadiazole drug-like substances. Compounds **9b** and **9g** were chosen to be encapsulated in Na-alginate microspheres. The release profile and mechanism of both compounds were investigated, and the results revealed that the release profiles of both microspheres showed a sustained release, and the release mechanism was controlled by Fickian diffusion. Accordingly, these compounds are promising for their use in chemotherapy for cancer treatment, and their hydrophilicity was improved by polymer encapsulation to become more effective in their pharmaceutical application.

## 1. Introduction

Cancer is one of the major causes of death today [1]. Usually, there are three main ways for treatment of cancer—Either individually or combined; surgery, chemotherapy, and radiation. Chemotherapy is a common method for cancer treatment [2].

Investigation of novel structures, designing and synthesis of some new, potent, and less toxic anti-cancer agents remains a major challenge for medical chemists.

The 1,3,4-Thiadiazoles- and 1,2,3-Triazole-based heterocycles have been well exploited for the production of many new medicinal scaffolds. While, the 1,3,4-Thiadiazole ring system is one of the most well-known important heterocyclic nuclei, as a core structural component, common and integral feature of a variety of biomedical agents and natural products. They attract considerable interest owing to their wide spectrum of biological and pharmaceutical applications. They have anti-tuberculosis, antibacterial, antihepatitis B viral, antifungal, antileishmanial, anti-inflammatory, CNS depressant, analgesic, anticancer, antioxidant, diuretic, molluscicidal, antihypertensive, analgesic, antimicrobial, antidiabetic, antitubercular, and anticonvulsant activities [3,4,5,6,7,8,9].

In applied synthesis, 1,2,3-triazoles become useful as building blocks and are additionally important due to their several therapeutically interesting activities [10,11], including *β*3-selective adrenergic receptor agonists [12], antimicrobial agents [13], anti-HIV compounds [14,15,16,17], kinase inhibitors [18,19], other enzymes inhibitors [20,21], cephalosporin cefatrizine [22], and *β*-lactam antibiotic tazobactam [23]. They are also stable in relation to light, oxygen, moisture and metabolic process [24,25].

Environmental protection and waste prevention have become the main requirements in an overcrowded world of demands, in order to ensure a healthy life free, from contaminants. Green chemistry techniques have proven their efficiency in terms of environmental impact using environmentally benign reactions. It has been conclusively proven that green reactions were preferred over the conventional thermal reactions that have originated from their ease of handling, rapidity, purities of products, enhancement in yields, and simplicity of the green methods compared with the thermal methods. One of these techniques is grinding reactions, in which organic reactions were done under solvent-free conditions [26]. Motivated by the statement; “The best solvent is no solvent [27]”, we report here our solvent-free protocol for synthesis of new heterocycles of pharmaceutical and biochemical interest. Thus, some of the exothermic reactions could be achieved in excellent yields via grinding of the solids together or (solid/liquid) using the mortar pestle technique, known as grindstone chemistry. The reaction begins with grinding of the solids together with transfer of a small amount of energy by friction. Based on this simple technique, we synthesized a series of bioactive heterocycles.

As mentioned above, 1,3,4-thiadiazole compounds and their derivatives have showed potential therapeutic effect on different diseases. Nevertheless, the synthesis of therapeutic 1,3,4-thiadiazole derivatives, bearing 1,2,3-triazole moiety, by using grinding method, is considered a challenging issue.

Despite the previously mentioned wide spectrum biomedical and pharmaceutical application of 1,3,4-thiadiazole and 1,2,3-triazole compounds, heterocyclic compounds in general are poorly soluble in water, and hence, have poor bioavailability, and they almost removed from the blood stream quickly without performing their function, only a small fraction of the administered doses reached the circulatory system and hence the target organ. Consequently, they are preferred to be delivered to the tumor cells by attaching to a hydrophilic carrier to increase their bio-accessibility and chemo-sensitivity [28]. The common carriers for delivering water insoluble drugs are hydrophilic polymer carriers, such as alginate polymer. Alginate is a biocompatible natural hydrophilic polysaccharide polymer isolated from marine brown algae. It has been reported that it increases the wettability of hydrophobic drug molecules [29,30], and accordingly, it has been used successful in delivering and encapsulating thiadiazole-based compounds [31,32,33,34]. Generally, the encapsulation of drugs with polymers is one of the versatile methods in controlling drug release over a long period of time and solves the complications related to traditional drug administration methods in chemotherapy. Moreover, this encapsulation can decrease or diminish the toxicity of therapeutic molecules, resulting from their high released concentrations in the case of conventional administration methods. One of the versatile methods for drug encapsulation forms is the biodegradable polymer microspheres. The preparation of microspheres is easy, cost effective, and it has little environmental impact. Moreover, polymeric microspheres can encapsulate most kinds of drugs, from water soluble to water insoluble drugs.

This work aimed mainly to prepare a new series of potent anti-cancer compounds, using grinding solvent-free reactions, which are considered to be of special efficacy with serious simplifications and improvements over the conventional methods. In addition, two of these synthesized compounds were encapsulated in alginate polymer in a microsphere form to improve their release and therapeutic potential. The hydrophobic defect of these compounds was solved by their encapsulation in hydrophilic Na-alginate polymer microspheres formulation. The drug release profile from the microspheres and the release mechanism were assessed.

## 2. Results and Discussion

### 2.1. Chemistry

**Scheme 1:**
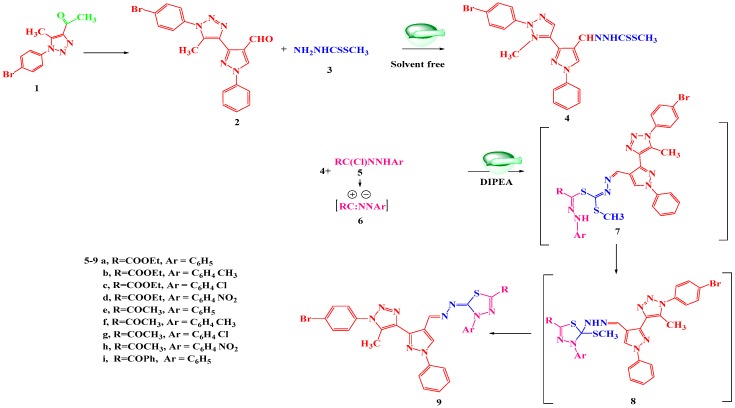
The mechanism for the formation of **9a**–**i**.

Condensation of 3-(1-(4-bromophenyl)-5-methyl-1*H*-1,2,3-triazol-4-yl)-1-phenyl-1*H*-pyrazole-4-carbaldehyde (**2**) with methyl hydrazinecarbodithioate (**3**) by grinding method at room temperature afforded the desired compound methyl 2-((3-(1-(4-bromophenyl)-5-methyl-1*H*-1,2,3-triazol-4-yl)-1-phenyl-1*H*-pyrazol-4-yl)methylene)hydrazine-1-carbodithioate (**4**). Structure of **4** was inferred from its spectral and micro-analytical data, its IR spectrum revealed the presence of the characteristic band for NH group and found it devoid of any bands for carbonyl group of CHO. In addition, its ^1^H NMR spectrum recorded a singlet signal at δ = 13.34 ppm for NH. Also, the structure was supported by mass spectra, which agree with the molecular formula (see Experimental part). The chemical composition of **4** was also confirmed via chemical transformation. Where, it was reacted with a series of selected derivatives of hydrazonoyl halides by grinding in the presence of 2–3 drops of DIPEA (*N*,*N*-Diisopropylethylamine) to give products assigned as 1-5-(3-(1-(4-bromophenyl)-5-methyl-1*H*-1,2,3-triazol-4-yl)-1-phenyl-1*H*-pyrazol-4yl)methylene)hydrazono)-4-(substituted)-4,5-dihydro-1,3,4-thiadiazol-2-yl) derivatives (**9a**–**i**) (Scheme 1) based on their spectral and micro-analytical data. The IR spectra of **9a**–**i** revealed absorption bands at 1680–1730 cm^−1^ owing to the carbonyl stretching bands while the absorption bands of the NH groups were disappeared. ^1^H NMR spectrum of **9a** for example showed signals at δ = 1.32 (t, 3H, *J* = 7.5 Hz, CH_2_**C**H_3_), 4.35 (q, 2H, *J* = 7.5 Hz, **CH_2_**CH_3_). Furthermore, ^13^C NMR spectrum of **9a** showed signals at δ = 14.43 (CH_3_), 63.20 (CH_2_), 118.26–149.85 (Ar-C), 158.48 ppm (C=O). ^1^H NMR spectrum of **9f** showed signals at δ = 2.37 (s, 3H, CH_3_), 2.51(s, 3H, CH_3_). Its ^13^C NMR spectrum showed signals at δ = 21.12 (CH_3_), 25.45 (CH_3_), 118.31–144.34 (Ar-C), 165.5 ppm (C=O) (see Experimental part).

Focusing on the preceding results, the mechanism outlined in Scheme 1 seems to be the most acceptable pathway for the formation of **9a**–**i**. The reaction involved the initial formation of thiohydrazonate **7**, which submitted to intermolecular cyclization as soon as it was formed to yield the intermediate **8** or through 1,3-dipolar cycloaddition of nitrilimine **6** (which was prepared in situ from **5** with DIPEA) to the double bond (C=S) of **4**. Finally, compound **8** was converted to **9** via the elimination of the methyl mercaptan group.

### 2.2. Anti-Cancer Activity

**Figure 1:**
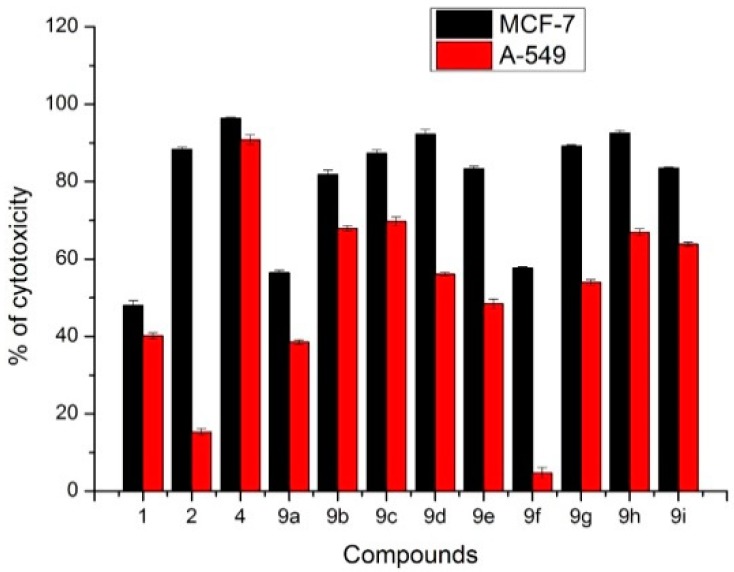
Screening the cytotoxic activities of compounds, in vitro, against human tumor cell lines: breast carcinoma (MCF-7) and human lung carcinoma (A-549 cell line). Preliminary concentration for screening was 100 ppm. Each result is a mean of 3 replicate samples.

**Figure 2:**
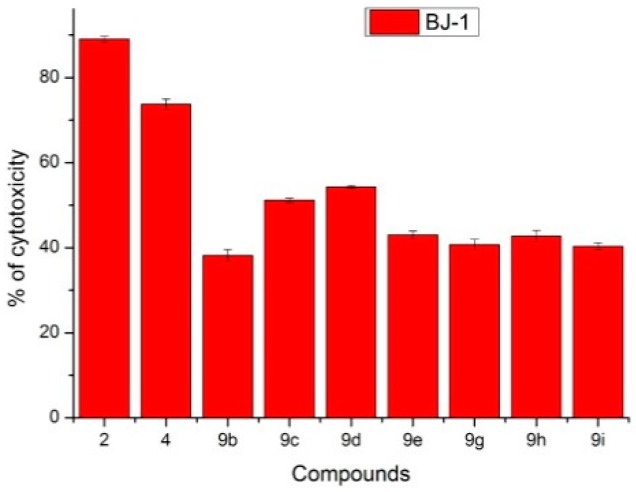
Screening the cytotoxic activities of active compounds, in vitro, against skin human normal cell line (BJ-1). Preliminary concentration for screening was 100 ppm. Each result is a mean of 3 replicate samples.

A total of 12 compounds were screened for their cytotoxic potentiality over human breast carcinoma (MCF-7 cell line) and human lung carcinoma (A-549 cell line) as shown in Figure 1. Compounds **4**, **9h**, **9d**, **9g**, **2**, **9c**, **9i**, **9e**, and **9b**, showed remarkable anti-cancer activity on breast cells with 96.4, 92.6, 92.3, 89.2, 88.4, 87.3, 83.5, 83.4, and 81.9% of cytotoxic activity, respectively. While compound **4** showed the most anti-cancer activity on lung cells with 90.8%. Afterwards, these compounds, which possessed ≥80% anti-cancer activity over breast and lung cells, were subjected to testing of their safety on human normal cell line (BJ-1) (Figure 2). Compound **9b**, **9i** and **9g** possessed high specificity with minimum toxicity on normal skin cells with 38.2, 40.3, and 40.7 of toxicity, respectively.

Compounds **9b** and **9g** were chosen due to their high breast anticancer activity and their safety in elucidating the possible anticancer mode of action.

**Figure 3:**
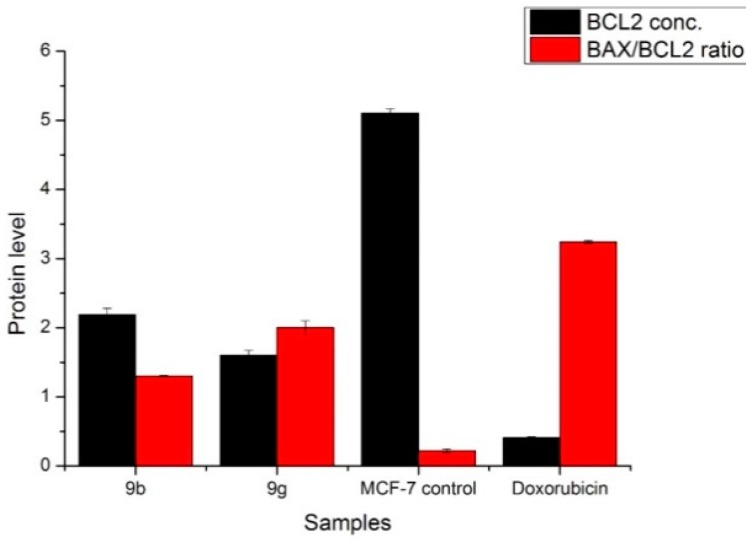
Bcl2 protein level (ng/mL) and BAX/BCL2 ratio of MCF-7treated with IC_50_ of compound **9b** (19.8 µg/mL) and IC_50_ of **9g** (26.2 µg/mL) compared to untreated cells and cells treated with reference drug Doxorubicin. *p* < 0.05 in **9b** and **9g**.

Bcl2 family protein included the anti-apoptotic protein Bcl-2 and proapoptotic protein Bax, which have been reported to regulate the induction of intrinsic apoptosis through mitochondria. Breast cancer cells were treated with compound **9b** and **9g** at different concentrations to determine the LC_50_ value of compound **9b** and **9g**. Afterwards, BCL2, BAX and BAX/BCL2 ratios were estimated by treated breast cells with 19.8 and 26.2 µg/mL” the LC_50_ value” of compound **9b** and **9g** respectively. Compound **9b** and **9g** possessed upregulate of BAX protein level and downregulate of BCL2 protein level with statistically significant (*p* < 0.05). As well, compound **9b** and **9g** disturb the ratio BAX/BCL2, which signal the apoptotic cascade inside the cells (Figure 3) and (Table 1).

**Figure 4:**
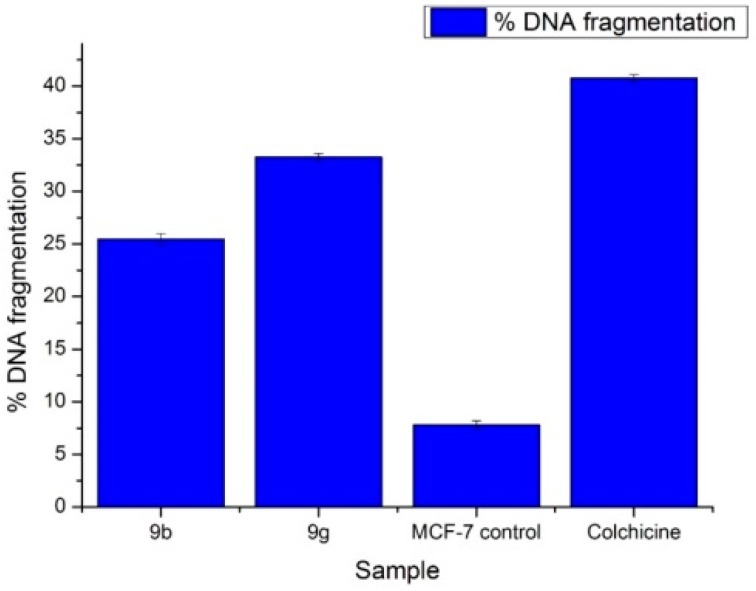
The percentage DNA fragmentation of treated cells with IC_50_ of compound **9b** (19.8 µg/mL) and IC_50_ of **9g** (26.2 µg/mL) compared to untreated control cells and cells treated with reference drug Colchicine (*p* < 0.05).

**Figure 5:**
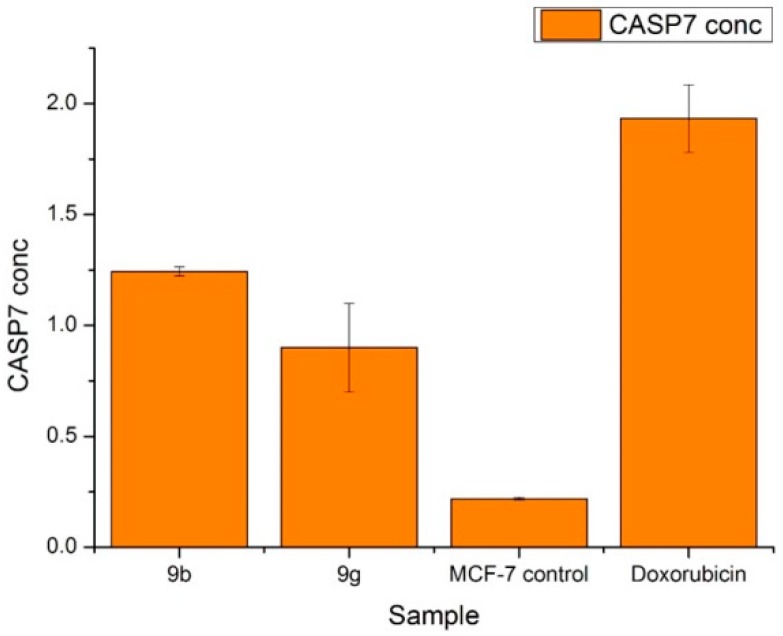
Casp7 (ng/mL) quantitative estimation of MCF-7 treated with IC_50_ of compound **9b** (19.8 µg/mL) and IC_50_ of **9g** (26.2 µg/mL) compared to untreated cells and cells treated with reference to the drug Doxorubicin (*p* < 0.05).

The effect of compounds **9b** and **9g** on DNA fragmentation were studied as well, MCF-7 was treated with 19.8 and 26.2 µg/mL of compound **9b,** and **9g** respectively and % of DNA fragmentation were determined (Figure 4). Compound **9b** and **9g** upregulate the protein level of caspase 7 in comparison with untreated cells with significant difference (*p* < 0.05). While, **9b** and **9g** induce apoptotic cascades inside cells through the activation of effector caspase 7 protein (Figure 5).

### 2.3. Microspheres Encapsulation

Compounds **9b** and **9g** were chosen as examples to be encapsulated in Na-alginate microspheres, the samples were encoded after encapsulation as **M9b**, and **M9g**, respectively and the blank microspheres encoded as **B**.

### 2.4. Morphology and Size of Microspheres

**Figure 6:**
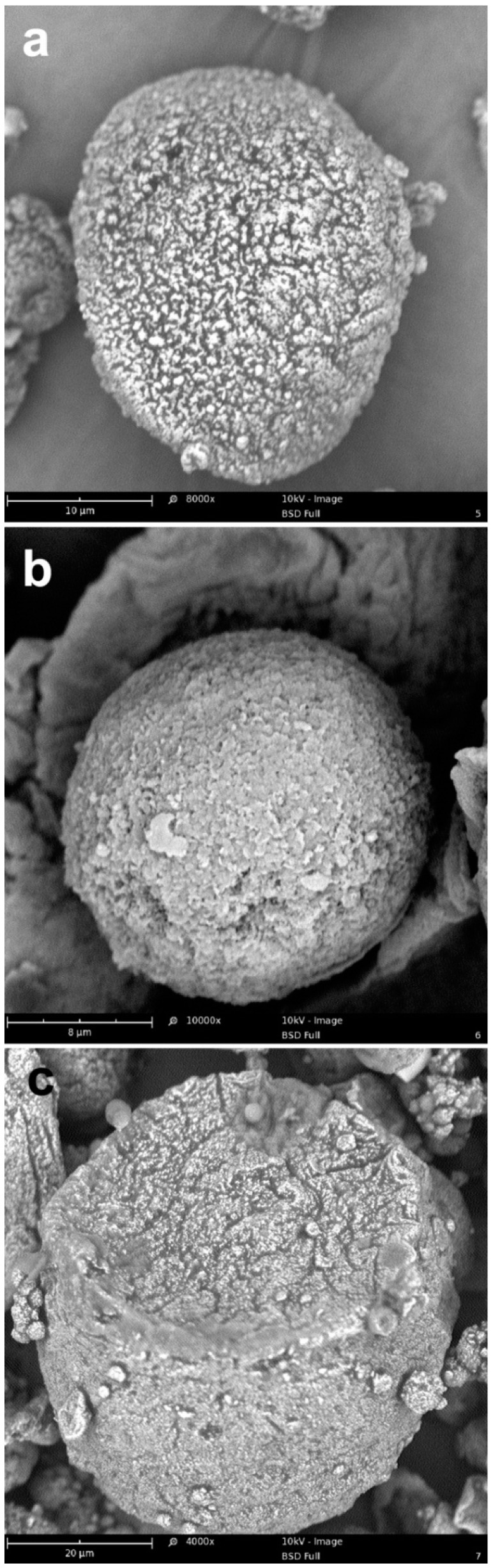
SEM micrographs of **B** microspheres (**a**), **M9b** (**b**) and **M9g** (**c**).

The morphology and diameter of prepared microspheres were determined from SEM micrographs (Figure 6). As shown in the Figure 6, the microspheres were spherical with rough surfaces. Moreover, the mean diameter values of microspheres, measured from SEM micrographs, were 38 µm, 24 µm, and 47 µm for **B**, **M9b**, and **M9g** samples, respectively. As a consequence, **M9b** microspheres showed the smallest diameters, while **M9g** microspheres demonstrated the biggest ones. This can be attributed to the chemical composition of the encapsulated heterocyclic compounds. The chemical structure of **9b** contained the Cl atom, which is highly electronegative. As a result, there was a likely repulsion force generated among carboxylic and hydroxyl groups in the polymer chains and the Cl^−^ ions in **9g** compound.

### 2.5. Thermal Gravimetric Analysis (TGA)

**Figure 7:**
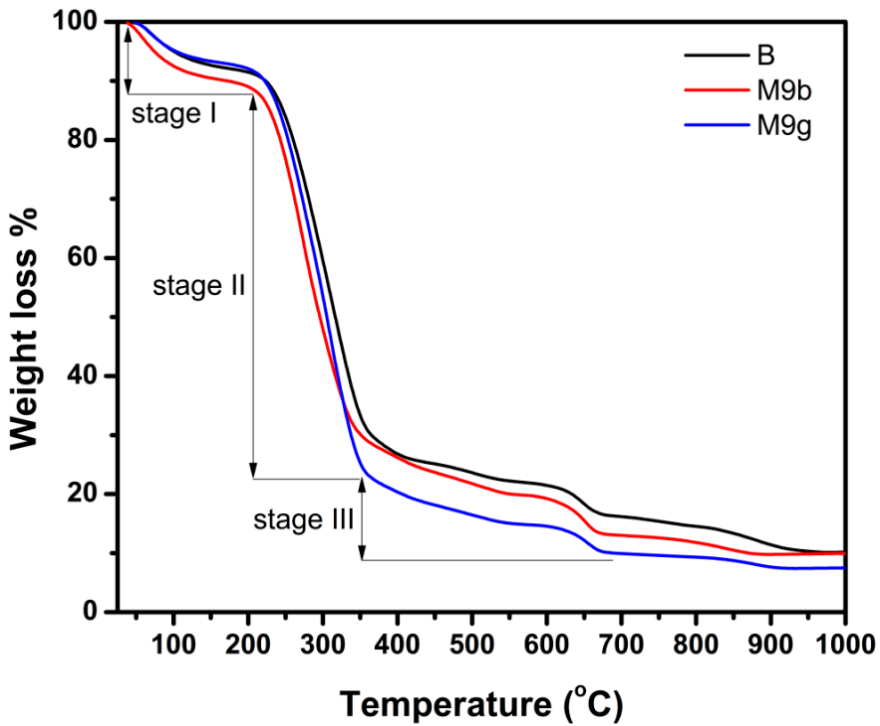
Thermal gravimetric analysis (**TGA**) analysis of **B**, **M9b** and **M9g** microspheres.

Figure 7 represents the TGA curves of **B**, **M9b**, and **M9g** samples. All the curves of different microspheres showed three weight loss stages. The first on (stage I) was observed before 200 °C, which can be attributed to the elimination of water molecules, adsorbed on the polymeric microspheres. The second one (stage II) was observed between 200 °C and 350 °C. This weight loss was assigned to thermal decomposition of the polymer. The third weight loss (stage III) was observed from 350 °C to 680 °C, due to the complete decomposition of alginate polymer. Remarkably, the percentages of weight losses of **M9b** and **M9g** microspheres were higher than that of blank microspheres (sample **B**), which was evident during stage III. This was explained by the removal and decomposition of encapsulated **9b** and **9g** chemical compounds.

### 2.6. Antitumor Activity of **M9b** and **M9g** over Breast Cancer Cell Line (MCF-7)

**M9b** and **M9g** were screened for their cytotoxic potentiality on the breast cancer cell line (MCF-7) on the same concentration used in their corresponding compounds **9b** and **9g**. Both **M9b** and **M9g** showed moderate activity 48.4, and 45.7%, respectively, after 48 h treatment. Meanwhile, **M9b** and **M9g** showed no toxicity on normal cell line BJ-1, which refer to the safety and selectivity of **M9b** and **M9g**.

### 2.7. Drug Release Profile and Kinetics

**Figure 8:**
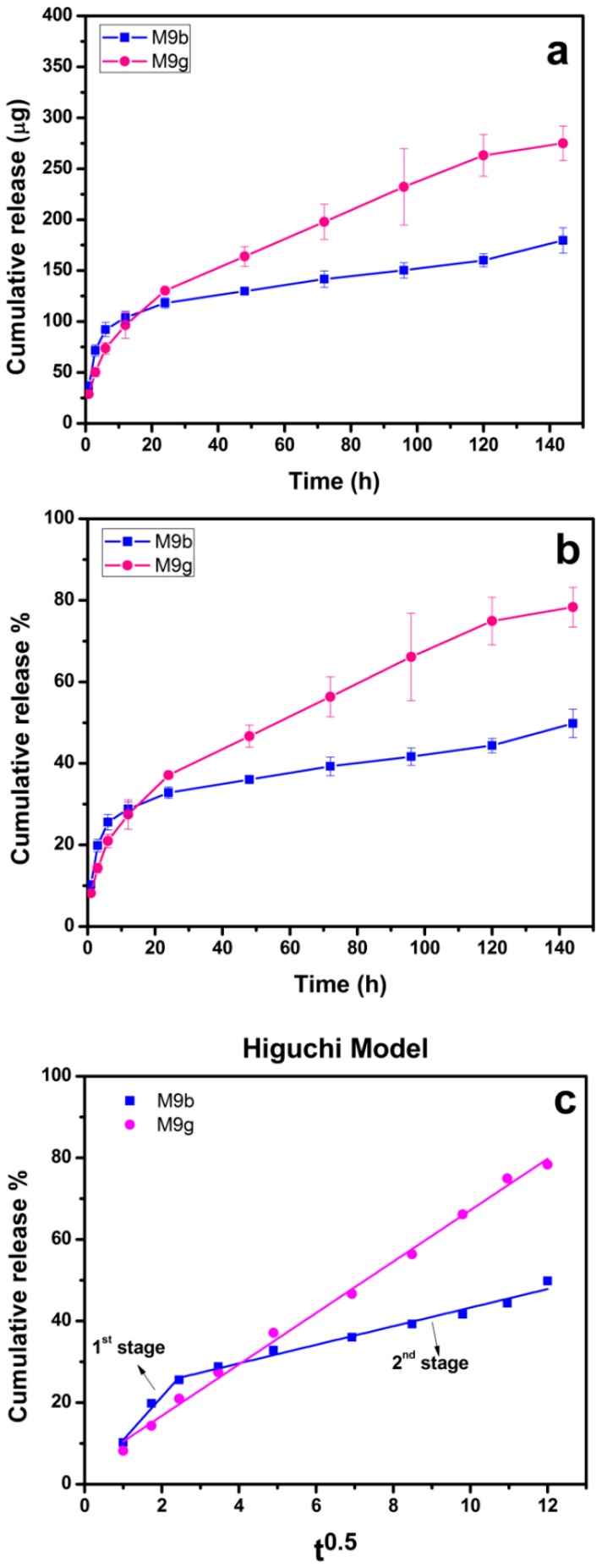
(**a**) Cumulative release amount (µg), (**b**) cumulative release %, and (**c**) fitting of release data with Higuchi model for **M9b** and **M9g** microspheres.

The amounts of encapsulated chemical compounds were approximately equal, the values were; 7.8 µg/mg and 7.0 µg/mg of **M9b**, and **M9g** microspheres, respectively. However, a slightly lower encapsulated amount of **9g** in alginate microsphere was likely to be attributed to the presence of Cl atom in the chemical structure of compound **9g**, which increased the electronegativity and hence the repulsion among it, and carboxyl and hydroxyl groups in alginate chains. Figure 8a,b show the amounts (µg), and the percentages of released drugs, respectively, for **M9b** and **M9g** samples. Generally, the release profiles of **M9b** and **M9g** microspheres showed a sustained release in two stages. The first release stage was fast and occurred during the initial 6 h of immersion, while the second one was slow and occurred throughout the rest of the incubation period (from 6 h to 144 h). The amounts of released molecules after 6 h of immersion were 104 ± 6.9 and 96 ± 5.7 µg, and the percentages were 26 ± 1.9% and 21 ± 1.6% form **M9b**, and **M9g**, respectively. In contrast, the released amounts at the end of incubation time were 180 ± 12.5 µg and 275 ± 17.1 µg, and the percentages were 50 ± 3.5% and 78 ± 4.9% form **M9b,** and **M9g**, respectively. According to the cell viability tests, the initial released amounts of both compounds **M9b** and **M9g** were considered effective and the potential to diminish the cancer cells under investigation (human breast carcinoma (MCF-7 cell line)).

The mechanism of drug release was investigated by linear fitting of the data with Higuchi model (Figure 8c). The degree of linearity was determined from regression coefficient (R^2^), Higuchi constant, k (h^−0.5^) calculated as well. Form the figure it was noted that there were two release rates of **M9b** sample. The first one was fast release with k = 10.59 h^−0.5^ (R^2^ = 0.961), which occurred during the first 6 h of immersion, while the second one was slow release and occurred throughout the rest of incubation time with k = 2.27 h^−0.5^ (R^2^ = 0.978). In contrast, **M9g** was released in one step with k = 6.30 h^−0.5^ (R^2^ = 0.995). Accordingly, the release rate of the chemical compounds depended on the chemical structure of both compounds. Furthermore, the high value of R^2^ for all fittings indicated that the release mechanism was controlled by Fickian diffusion.

## 3. Experimental

### 3.1. Chemistry

#### Experimental Instrumentation

All melting points were determined on an electrothermal apparatus and are uncorrected. IR spectra were recorded (KBr discs) on a Shimadzu FT-IR 8201 PC spectrophotometer. ^1^H NMR and ^13^C NMR spectra were recorded in (CD_3_)_2_SO solutions on BRUKER 400 FT-NMR system spectrometer and chemical shifts are expressed in ppm units using TMS as an internal reference. Mass spectra were recorded on a GC-MS QP1000 EX Shimadzu. Elemental analyses were carried out at the Microanalytical Center of Cairo University. 1-(1-(4-bromophenyl)-5-methyl-1*H*-1,2,3-triazol-4-yl)ethanone (**1**) [35]. 3-(1-(4-bromophenyl)-5-methyl-1*H*-1,2,3-triazol-4-yl)-1-phenyl-1*H*-pyrazole-4-carbaldehyde (**2**) [11], methyl hydrazinecarbodithioate (**3**) [36] and hydrazonoyl halides **5a**–**i** [37,38,39] were prepared as previously reported.

### 3.2. Synthesis

#### 3.2.1. *Methyl 2-((3-(1-(4-bromophenyl)-5-methyl-1H-1,2,3-triazol-4-yl)-1-phenyl-1H-pyrazol-4-yl)methylene)hydrazine-1-carbodithioate* (**4**)

A mixture of 3-(1-(4-bromophenyl)-5-methyl-1*H*-1,2,3-triazol-4-yl)-1-phenyl-1*H*-pyrazole-4-carbaldehyde (**2**) and methyl hydrazinecarbodithioate (**3**) (5 mmol) was thoroughly ground with a pestle in an open mortar at room temperature for 3–5 min until the mixture turned into a melt. The initial syrup grinding proceeded for 5–10 min, and the reaction was monitored by TLC. The solid was washed with water and recrystallized from ethanol to give the corresponding methyl hydrazinecarbodithioate derivative **4**.

Grey Crystals, Yield: 92%, m.p.: 156–158 °C; FT-IR (KBr, cm^−1^): *v* 2982 (C–H), 3119 (NH), 1625 (C=N), 1612 (C=C); ^1^H NMR (400 MHz, DMSO-*d*_6_): δ 2.52 (s, 3H, CH_3_), 2.63 (s, 3H, CH_3_), 7.3–8.03 (m, 9H, Ar-H), 8.97 (s, 1H, CH=N), 8.98 (s, 1H, C-H pyrazole), 13.34 (s, 1H, NH); MS: *m*/*z* [%]: 511 (M^+^, 17), 496 (22), 450 (18), 378 (3), 273 (21), 250 (36), 177 (100), 165 (18), 77 (90), 55 (43). Analysis: calcd. For C_21_H_18_BrN_7_S_2_ (511): C, 49.22; H, 3.54; N, 19.13%. Found: C, 49.35; H, 3.45; N, 19.05%.

#### 3.2.2. General Procedures for Synthesis of **9a**–**i**

Methyl hydrazinecarbodithioate derivative **4** (2.5g, 5 mmol) and the appropriate hydrazonoyl halides **5a**–**i** (5 m mol) with the addition of 2–3 drops of DIPEA, were mixed and ground with a pestle in an open mortar at room temperature for 3–5 min until the mixture turned into a melt. The initial syrup grinding proceeded for 5–10 min, and the reaction was monitored by TLC (Thin Layer Chromatography). The solid was washed with water, then ethanol, and recrystallized from acetic acid to give **9a**–**i**, respectively.

*Ethyl 5-((-(3-(1-(4-bromophenyl)-5-methyl-1H-1,2,3-triazol-4-yl)-1-phenyl-1H-pyrazol-4-yl)methylene)hydrazono)-4-phenyl-4,5-dihydro-1,3,4-thiadiazole-2-carboxylate* (**9a**) Yellow Crystals, Yield: 89%, m.p.: 196–198 °C; FT-IR (KBr, cm^−1^): *v* 2987 (C–H), 1725 (C=O), 1630 (C=N), 1600 (C=C); ^1^H NMR (400 MHz, DMSO-*d*_6_): δ 1.32 (t, 3H, CH_2_**CH_3_**), 2.51 (s, 3H, CH_3_), 4.35 (q, 2H, CH_2_), 7.38–8.01 (m, 15H, Ar-H, CH=N), 9.02 (s, 1H, C-H pyrazole); ^13^C NMR (100 MHz, DMSO-*d*_6_): δ 10.34, 14.43, 63.20, 118.26, 119.17, 122.84, 123.37, 127.63, 129.54, 133.14, 135.40, 138.45, 139.02, 144.34, 149.85, 158.48; MS: *m*/*z* [%]: 656 (M+2, 37), 654 (50), 639 (12), 624 (9), 611 (18), 578 (5), 530(6), 482 (21), 430 (12), 377 (13), 278 (2), 265 (3), 178 (80), 160 (10), 66 (90), 50 (100). Analysis: calcd. For C_30_H_24_BrN_9_O_2_S (654): C, 55.05; H, 3.70; N, 19.26%. Found: C, 55.12; H, 3.62; N, 19.17%.

*Ethyl 5-((-(3-(1-(4-bromophenyl)-5-methyl-1H-1,2,3-triazol-4-yl)-1-phenyl-1H-pyrazol-4-yl)methylene)hydrazono)-4-(p-tolyl)-4,5-dihydro-1,3,4-thiadiazole-2-carboxylate* (**9b**). Yellow Crystals, Yield: 93%, m.p.: 205–207 °C; FT-IR (KBr, cm^−1^): *v* 2991 (C–H), 1727 (C=O), 1620 (C=N), 1610 (C=C); ^1^H NMR (400 MHz, DMSO-*d*_6_): δ 1.37 (t, 3H, CH_2_CH_3_), 2.58 (s, 3H, CH_3_), 2.64 (s, 3H, CH_3_), 4.22 (q, 2H, **CH_2_**CH_3_), 7.5–8.13 (m, 14H, Ar-H, CH=N), 9.12 (s, 1H, C-H pyrazole); MS: *m*/*z* [%]: 667 (M^+^, 12), 653 (15), 637 (1), 622 (9), 608 (22), 560 (15), 527(16), 482 (21), 430 (12), 270 (21), 267 (13), 176 (11), 166 (19), 70 (100). Analysis: calcd. For C_31_H_26_BrN_9_O_2_S (667): C, 55.69; H, 3.92; N, 18.86%. Found: C, 55.58; H, 3.81; N, 18.85%.

*Ethyl 5-((-(3-(1-(4-bromophenyl)-5-methyl-1H-1,2,3-triazol-4-yl)-1-phenyl-1H-pyrazol-4-yl)methylene)hydrazono)-4-(4-chlorophenyl)-4,5-dihydro-1,3,4-thiadiazole-2-carboxylate* (**9c**). Yellow Crystals, Yield: 85%, m.p.: 220–222 °C; FT-IR (KBr, cm^−1^): *v* 2982 (C–H), 1730 (C=O), 1622 (C=N), 1612 (C=C); ^1^H NMR (400 MHz, DMSO-*d*_6_): δ 1.33 (t, 3H, CH_2_**CH_3_**), 2.59 (s, 3H, CH_3_), 4.35 (q, 2H, **CH_2_**CH_3_), 7.15–8.95 (m, 13H, Ar-H), 8.97 (s, 1H, CH=N), 9.04 (s, 1H, C-H pyrazole); MS: *m*/*z* [%]: 687 (M^+^, 3), 642 (17), 612 (21), 610 (2), 572 (19), 532(6), 491 (11), 430 (12), 270 (21), 272 (43), 180 (18), 167 (19), 55 (100). Analysis: calcd. For C_30_H_23_BrClN_9_O_2_S (687): C, 52.30; H, 3.36; N, 18.30%. Found: C, 52.25; H, 3.31; N, 18.19%.

*Ethyl 5-(((3-(1-(4-bromophenyl)-5-methyl-1H-1,2,3-triazol-4-yl)-1-phenyl-1H-pyrazol-4-yl)methylene)hydrazono)-4-(4-nitrophenyl)-4,5-dihydro-1,3,4-thiadiazole-2-carboxylate* (**9d**). Yellow Crystals, Yield: 83%, m.p.: 255–257 °C; FT-IR (KBr, cm^−1^): *v* 2977 (C–H), 1727 (C=O), 1628 (C=N), 1600 (C=C); ^1^H NMR (400 MHz, DMSO-*d*_6_): δ 1.32 (t, 3H, CH_2_CH_3_), 2.56 (s, 3H, CH_3_), 4.30 (q, 2H, **CH_2_**CH_3_), 7.11–8.85 (m, 14H, Ar-H CH=N), 8.98 (s, 1H, C-H pyrazole); MS: *m*/*z* [%]: 699 (M^+^, 53), 672 (8), 651 (15), 640 (25), 602 (7), 600 (21), 565 (14), 537 (16), 480 (21), 412 (12), 278 (24), 266 (33), 186 (8), 177 (100), 65 (50). Analysis: calcd. For C_30_H_23_BrN_10_O_4_S (699): C, 51.51; H, 3.31; N, 20.02%. Found: C, 51.58; H, 3.27; N, 19.95%.

*1-(5-((-(3-(1-(4-Bromophenyl)-5-methyl-1H-1,2,3-triazol-4-yl)-1-phenyl-1H-pyrazol-4-yl)methylene)hydrazono)-4-phenyl-4,5-dihydro-1,3,4-thiadiazol-2-yl)ethan-1-one* (**9e**). Yellow Crystals, Yield: 91%, m.p.: 199–201 °C; FT-IR (KBr, cm^−1^): *v* 1685 (C=O), 1630 (C=N), 1597 (C=C); ^1^H NMR (400 MHz, DMSO-*d*_6_): 2.51 (s, 3H, CH_3_), 2.57 (s, 3H, CH_3_), 7.33–8.15 (m, 14H, Ar-H), 9.02 (s, 1H, CH=N), 9.05 (s, 1H, C-H pyrazole); MS: *m*/*z* [%]: 624 (M^+^, 27), 601 (18), 591 (5), 570 (21), 523 (17), 500 (14), 485 (4), 465 (6), 432 (2), 381 (9), 280 (3), 256 (33), 180 (18), 176 (100), 66 (30). Analysis: calcd. For C_29_H_22_BrN_9_OS (624): C, 55.77; H, 3.55; N, 20.19%. Found: C, 55.65; H, 3.42; N, 20.13%.

*1-(5-((-(3-(1-(4-bromophenyl)-5-methyl-1H-1,2,3-triazol-4-yl)-1-phenyl-1H-pyrazol-4-yl)methylene)hydrazono)-4-(p-tolyl)-4,5-dihydro-1,3,4-thiadiazol-2-yl)ethan-1-one* (**9f**). Yellow Crystals, Yield: 91%, m.p.: 285–287 °C; FT-IR (KBr, cm^−1^): *v* 1680 (C=O), 1635 (C=N), 1611 (C=C); ^1^H NMR (400 MHz, DMSO-*d*_6_): 2.37 (s, 3H, CH_3_), 2.51 (s, 3H, CH_3_), 2.63 (s, 3H, CH_3_), 7.37–8.05 (m, 13H, Ar-H), 9.02 (s, 1H, CH=N), 9.08 (s, 1H, C-H pyrazole); ^13^C NMR (100 MHz, DMSO-*d*_6_): δ 10.32, 21.12, 25.45, 118.31, 119.25, 123.10, 127.61, 130.01, 133.17, 135.41, 136.60, 138.41, 139.32, 144.34, 165.5; MS: *m*/*z* [%]: 624 (M^+^, 27), 601 (18), 591 (5), 570 (21), 523 (17), 500 (14), 485 (4), 465 (6), 432 (2), 381 (9), 280 (3), 256 (33), 180 (18), 176 (100), 66 (30). Analysis: calcd. For C_29_H_24_BrN_9_OS (637): C, 56.43; H, 3.79; N, 19.74%. Found: C, 56.32; H, 3.73; N, 19.65%.

*1-(5-((-(3-(1-(4-Bromophenyl)-5-methyl-1H-1,2,3-triazol-4-yl)-1-phenyl-1H-pyrazol-4-yl)methylene)hydrazono)-4-(4-chlorophenyl)-4,5-dihydro-1,3,4-thiadiazol-2-yl)ethan-1-one* (**9g**). Yellow Crystals, Yield: 89%, m.p.: 212–214 °C; FT-IR (KBr, cm^−1^): *v* 1686 (C=O), 1620 (C=N), 1601 (C=C); ^1^H NMR (400 MHz, DMSO-*d*_6_): 2.53 (s, 3H, CH_3_), 2.61 (s, 3H, CH_3_), 7.41–8.36 (m, 14H, Ar-H, CH=N), 9.08 (s, 1H, C-H pyrazole); MS: *m*/*z* [%]: 660 (M+2, 12), 658 (M^+^, 18), 632 (3), 605 (1), 590(15), 578 (31), 543 (19), 509 (25), 486 (14), 463 (16), 427 (12), 391 (91), 287 (32), 257 (30), 190 (8), 177 (100), 60 (52). Analysis: calcd. For C_29_H_21_BrClN_9_OS (658): C, 52.86; H, 3.21; N, 19.13%. Found: C, 52.93; H, 3.16; N, 19.02%.

*1-(5-((-(3-(1-(4-Bromophenyl)-5-methyl-1H-1,2,3-triazol-4-yl)-1-phenyl-1H-pyrazol-4-yl)methylene)hydrazono)-4-(4-nitrophenyl)-4,5-dihydro-1,3,4-thiadiazol-2-yl)ethan-1-one* (**9h**). Yellow Crystals, Yield: 95%, m.p.: 218–220 °C; FT-IR (KBr, cm^−1^): *v* 1680 (C=O), 1625 (C=N), 1600 (C=C); ^1^H NMR (400 MHz, DMSO-*d*_6_): 2.57 (s, 3H, CH_3_), 2.63 (s, 3H, CH_3_), 7.31–8.39 (m, 14H, Ar-H, CH=N), 8.96 (s, 1H, C-H pyrazole); MS: *m*/*z* [%]: 669 (M^+^, 28), 652 (30), 639 (21), 623 (9), 607 (6), 593 (2), 590 (35), 580 (14), 534 (29), 518 (2), 480 (41), 465 (61), 430 (12), 397 (1), 282 (23), 259 (32), 197 (18), 178 (90), 50 (100). Analysis: calcd. For C_29_H_21_BrN_10_O_3_S (669): C, 52.03; H, 3.16; N, 20.92%. Found: C, 52.12; H, 3.01; N, 20.87%.

*(-5-((-(3-(1-(4-bromophenyl)-5-methyl-1H-1,2,3-triazol-4-yl)-1-phenyl-1H-pyrazol-4-yl)methylene)hydrazono)-4-phenyl-4,5-dihydro-1,3,4-thiadiazol-2-yl)(phenyl)methanone* (**9i**). Brownish Yellow Crystals, Yield: 86%, m.p.: 230–232 °C; FT-IR (KBr, cm^−1^): v 1687 (C=O), 1633 (C=N), 1609 (C=C); ^1^H NMR (400 MHz, DMSO-*d*_6_): 2.53 (s, 3H, CH_3_), 7.17–8.98 (m, 19H, Ar-H), 9.08 (s, 1H, CH=N), 9.14 (s, 1H, C-H pyrazole); MS: *m*/*z* [%]: 686 (M^+^, 33), 671 (3), 592 (27), 525 (19), 459 (16), 393 (32), 281 (13), 266 (37), 195 (100), 177 (20), 55 (60). Analysis: calcd. For C_34_H_24_BrN_9_OS (686): C, 59.48; H, 3.16; 3.52 N, 18.36%. Found: C, 59.35; H, 3.13; N, 18.31%.

### 3.3. Anti-Cancer Activity

#### 3.3.1. Cell Lines

Human breast carcinoma (MCF-7 cell line), human lung carcinoma (A-549 cell line) and skin normal human cell line (BJ-1); “a telomerase immortalized normal foreskin fibroblast cell line” were obtained from Karolinska Center, Department of Oncology and Pathology, Karolinska Institute and Hospital, Stockholm, Sweden.

#### 3.3.2. Cell Culture

The procedure was carried out in a sterile area using a laminar air flow cabinet biosafety class II level. The culture was maintained in RPMI 1640 medium with 1% antibiotic-antimycotic mixture (10,000 U/mL potassium penicillin, 10,000 μg/mL streptomycin sulfate and 25 μg/mL amphotericin B), 1% L-glutamine, and supplemented with 10% heat inactivated fetal bovine serum. Culturing and sub culturing were carried out according to Thabrew et al. [40]. Doxorubicin was used as a positive control. A negative control, composed of DMSO, was also used.

#### 3.3.3. Cell Viability Assay

This was done according to Selim et al. [41], as described by Mosmann [42]. The cells were seeded at concentration of 10 × 10^3^cells per well in case of MCF-7, 20 × 10^3^ cells/well in case of A-549 cell lines and 35–45 × 10^3^ cells/well in case of BJ-1 using 96-well plates at 37 °C. After 48 h of incubation, the medium was aspirated and 40 μL MTT salt (2.5 mg/mL) were added and further incubated for 4 h. Then, 200 μL 10% sodium dodecyl sulphate (SDS) was added. The absorbance was measured at 595 nm.

#### 3.3.4. Determination of IC_50_ Values

IC_50_ values were calculated, using a probit analysis, and by utilizing the SPSS computer program (SPSS for windows, statistical analysis software package/version 9/1989 SPSS Inc., Chicago, IL, USA).

#### 3.3.5. Measurement of DNA Fragmentation using DPA Assay

DNA fragmentation of the cells was assayed, as described by Cohen and Duke & Burton [43,44]. Briefly, the cells were lysed with lysis buffer containing 20 mM EDTA, 0.5% (*v*/*v*) Triton X-100, and 5 mivi Tris (pH 8.0) for 15 min on ice. The cells were then centrifuged for 20 min, at 27,000× *g*, to separate intact chromatin (pellet) from DNA fragments (supernatant). The amount of DNA in both the pellet and the supernatant was measured by using a diphenylamine reagent. The optical density is a colorimetric, measured at 600 nm. The percentage of DNA fragmentation was calculated as the ratio of DNA in supernatant and DNA in pellet.

#### 3.3.6. Human CASP7 (Caspase 7) Estimation

The micro ELISA plate provided in this kit pre-coated with CASP7 specific antibody. A biotinylated CASP7 antibody and Avidin-Horseradish Peroxidase (HRP) conjugate was added. The excess components were aspired. The substrate solution was then added. The wells, which contained CASP7, biotinylated detection antibody and Avidin-HRP conjugate will appear blue in color. The color turned yellow following the addition of sulfuric acid solution. The optical density (OD) was measured at a wavelength of 450 nm ± 2 nm [45].

#### 3.3.7. Measurement of BCl-2 Levels

BCL-2 in the samples and standards were estimated according to Barbareschi et al. [46]. A biotin-conjugated antibody was added followed by streptavidin-HRP. The reaction is then terminated by adding acid and absorbance was measured at 450 nm.

#### 3.3.8. Measurement of Bax Levels

Bax protein levels were evaluated according to Onur et al. [47]. Monoclonal antibody, specific to Bax captured on the plate, was added. After incubation, Streptavidin conjugated to Horseradish peroxidase was added. The reaction was then terminated by adding acid and optical density of the color produced measured at 450 nm [47].

### 3.4. Encapsulation in Na-alginate Microspheres

Microspheres, encapsulated by compounds **9b** and **9g**, were prepared by w/o emulsion and internal gelation method [48]. For microsphere synthesis, the following method was carried out; 5% (*w*/*v*) sodium alginate (from Sigma company, New Delhi, India) solution was prepared by dissolution of the polymer in distilled water. The solutions of both compounds **9b** and **9g** were prepared by dissolving them separately in DMSO solvent with a concentration of 10 mg/mL. After the complete dissolution of polymers, the compounds **9b** and **9g** were added to the polymer solution individually with volume ratio 1:4 (the microsphere samples were coded as **M9b** and **M9g**). Thereafter, 10% of Span^®^ 85 and paraffin oil (five times of the total volume) were added to the above solutions and stirred at 1000 rpm at 70 °C for 15 min, and then CaCl_2_ of 2 M concentration was added to the mixtures with volume ratio 1:5 and stirred for an additional 15 min at the same stirring speed and temperature. The solutions were suddenly cooled at 4 °C. The obtained microspheres were washed with isopropanol several times, to remove the paraffin oil, centrifuged and dried. For comparison, the microspheres without chemical compounds were prepared following the above protocol (coded as B). The morphology and thermal behavior of microspheres were determined by SEM (HITACHI SU 8000, Tokyo, Japan), and TGA (TGA Q500), respectively. On the other hand, the amounts of encapsulated **9b** and **9g** in alginate microspheres were determined by dissolving 10 mg of microspheres in 5 mL DMSO and the concentrations of **9b** and **9g** were determined by UV-VIS spectroscopy.

### 3.5. In Vitro Release Test

The release behavior of the synthetic chemical compounds encapsulated in Na-alginate microspheres (samples **M9b** and **M9g**) was investigated by immersing 50 mg of microspheres in 4.5 mL of phosphate buffer saline (PBS) with pH 7.4. The drug release was evaluated at 37 °C up to 6 days. At each predetermined time (1, 3, 6, 12 h, 1, 2, 3, 4, 5, and 6 d), 1.5 mL of immersed solution was collected and replaced by 1.5 mL fresh one. The collected solutions were kept at −20 °C up until the measurement. The concentrations of released drug were determined by measuring the absorbance by UV/VIS spectroscopy at wavelength 355 nm and plotting it on the standard curve. This wavelength was determined from the maxima in UV/VIS absorption curve using Jasco.

### 3.6. Drug Releasing Mechanism

The drug release mechanism was investigated by fitting the drug release profiles to the Higuchi model. This model was used extensively to define the diffusion controlled processes of released drug from the porous carrier [49]. This model is given by the following equation:

Q = k.t^0.5^ (1)

where, Q is the percentage of drug released at time t, and k is the release rate constant.

### 3.7. Statistical Analysis

All experimental data stated in this work were expressed as the average ± standard deviation (SD) for n = 3 and were analyzed using standard analysis of Student’s t-test. The level of significance (*p*-value) is set at < 0.05.

## 4. Conclusions

In this described investigation, a series of 1,3,4-Thiadiazole derivatives bearing 1,2,3-triazole moiety, were introduced as a new class of anti-cancer agents. The scaffolds have the advantage of synthetic protocol approachability.

Briefly, our target compounds were prepared through reaction of methyl 2-((3-(1-(4-bromophenyl)-5-methyl-1*H*-1,2,3-triazol-4-yl)-1-phenyl-1*H*-pyrazol-4-yl)methylene)hydrazine-1-carbodithioate (**4**) with selective derivatives of hydrazonoyl halides via eco-friendly grinding clean method under solvent free conditions. Compounds **9b** and **9g** have anti-cancer effects on breast cancer with safety effects on normal cells. Compound **9b** and **9g** induce apoptosis inside the cells by disturbing the balance between pro-apoptotic and anti-apoptotic protein, and increase the percentage DNA fragmentation, which triggers signals to activate Caspase 7. In turn this plays an executionary role in apoptosis. Furthermore, **9b** and **9g** were chosen to be encapsulated in Na-alginate microspheres, the encapsulation of selected compounds in alginate microspheres controlled their release, so that the release sustained, and the release mechanism was controlled by Fickian diffusion.

Accordingly, this work introduced new promising anti-cancer agents for chemotherapy, and solved the defects associated with their water poor solubility, and hence, poor bioavailability, by encapsulating in hydrophilic Na-alginate microspheres to increase the hydrophilicity. This formulation controlled the release of chemical compounds into the solution to a sustained release.

## Figures and Tables

**Table 1:** BAX protein level (Pg/mL) of MCF-7treated with IC_50_ of compound **9b** (19.8 µg/mL) and IC_50_ of **9g** (26.2 µg/mL) compared to untreated cells and cells treated with reference drug Doxorubicin.

	BAX CONC.
**9b**	160.5
**9g**	241.7
cont.	40.743
Doxorubicin	259.2
